# Positive effects of forest fragmentation *per se* on bryophyte diversity in subtropical fragmented forests: evidence from land-bridge islands

**DOI:** 10.3389/fpls.2025.1539513

**Published:** 2025-04-10

**Authors:** Dandan Li, Tonghe Yuan, Jun Yang, Shan Lv, Heng Zhang, Yuzhu Xia, Xiao Wang, Shuiliang Guo, Jing Yu

**Affiliations:** College of Life Sciences, Shanghai Normal University, Shanghai, China

**Keywords:** bryophytes, fragmentation *per se*, landscape, species richness, subtropical secondary forest

## Abstract

**Introduction:**

Habitat fragmentation (*Sensu lato*) represents a landscape-scale process involving both habitat loss and the breaking apart of habitat (habitat fragmentation *per se*). In ecological studies, understanding the impacts of habitat fragmentation *per se* on biodiversity remains a critical challenge. While previous research has explored the effects of fragmentation on various ecosystems, significant gaps remain in our understanding of its impacts on bryophyte assemblages.

**Methods:**

To explore the effects of habitat fragmentation *per se* on bryophyte assemblages in subtropical forests, we investigated bryophytes and environments on 18 fragmented forest landscapes (including 166 islands) in Thousand Island Lake, China. Landscape-level environmental variables of habitat fragmentation *per se* included island number, mean area, area variability, shape irregularity, shape variability, and isolation degree. Landscape-level habitat amount was represented by island total area within the study landscape. We investigated species richness (SR) and coverage in edge zones and interior environments of thirteen islands to explore the edge effects of fragmented forests on bryophytes.

**Results and discussion:**

Variance partitioning revealed that habitat fragmentation *per se* independently explained 38.92% of variation in bryophyte SR and 36.5% of variation in species composition (SC). Landscape-level Island total area explained 6.2% of SR variation and 5.9% of SC variation. Among the environmental variables associated with fragmentation *per se*, island number and shape irregularity were identified as the most significant, independently explaining 16.2% and 15.5% of variation in bryophyte SR, respectively. Island shape variability and area variability independently explained 5.3% and 2.1% variations in bryophyte SR, respectively. A linear increase in bryophyte SR was observed with island mean area and shape irregularity, while a nonlinear relationship was detected with island number, island shape irregularity and area variability. Island area variability, shape variability and island number influenced bryophyte SC to similar extents, independently explaining 5.9% to 6.6% of variation in bryophyte SC. Consequently, habitat fragmentation *per se* had pronounced effects on both bryophyte SR and SC in subtropical fragmented forests. Such effects were likely due to the positive edge effects of fragmented forests on bryophyte assemblages. Our findings suggest that, in subtropical fragmented secondary forests, the reserve for bryophytes had better contain numerous forest patches with irregular shapes, large total area, and moderate variation in island shape and area.

## 1 Introduction

Global landscapes are being fragmented at an alarming rate (Mullu, 2016). Habitat fragmentation (*Sensu lato, s.l.*) has been recognized to have profound negative impacts on biodiversity, thereby emerging as a defining feature of habitat degradation (Haila, 2002). This process remains a central concern in contemporary conservation biology (Bogaert et al., 2011).

Traditionally, habitat fragmentation (s*.l.*) was defined as a landscape-scale process encompassing both habitat loss and habitat fragmentation *per se* (a change in the spatial configuration of the remaining habitat for a given habitat amount) (Fahrig, 2003; Fahrig, 2013). Habitat fragmentation *per se* can be characterized by three primary components: an increase in the number of patches, a reduction in mean patch size, and enhanced patch isolation. Habitat loss and fragmentation *per se* may influence biodiversity through different mechanisms, thus these two process often require separate analytical approaches (Bird and Fahrig, 2013). However, most previous researches conflated the effects of habitat fragmentation *per se* with those of habitat loss (Bird and Fahrig, 2013). This conflation is problematic because the negative impacts attributed to habitat fragmentation (*s.l.*)) may actually reflect the effects of habitat loss, which consistently demonstrates strong negative impacts on biodiversity (Fahrig, 2003). Consequently, many studies examining the effects of habitat fragmentation (*s.l.*) on biodiversity have focused predominantly on habitat loss at the patch scale (Gignac and Dale, 2005; Panitsa et al., 2010; Bird and Fahrig, 2013; Wu et al., 2013), thereby obscuring the independent effects of fragmentation *per se* on biodiversity. Distinguishing between the independent effects of habitat loss and fragmentation *per se* is critical for optimizing the allocation of limited conservation resources (Palmeirim et al., 2019).

Recent studies have produced conflicting conclusions regarding the effects of habitat fragmentation *per se* on biodiversity. Some theoretical studies have argued that the direct effects of fragmentation *per se* were weaker than those of habitat loss (Collingham and Huntley, 2000; Fahrig, 1997; Flather and Bevers, 2002), but other studies predicted more pronounced effects of fragmentation *per se* (Boswell et al., 1998; Burkey, 1999; Hill and Caswell, 1999; Urban and Keitt, 2001; Fahrig, 2002). Fahrig (2017) analyzed 381 study cases that examined the independent effects of habitat fragmentation *per se* and found that 76% reported positive effects of fragmentation *per se* on biodiversity, regardless of methodological approaches to controlling variables such as habitat amount, the measure of fragmentation, and the taxonomic group. However, Fletcher et al. (2018) challenged this conclusion, asserting that Fahrig’s findings were based on a narrow and potentially biased subset of evidence, rather than the broader observational, experimental, and theoretical evidence indicating the negative effects of habitat configuration changes. In response, Fahrig et al. (2019) maintained that Fletcher Jr et al.’s argument was largely grounded in patch-scale rather than landscape-scale analyses. According to Rybicki et al. (2020), when the total amount of habitat is large, fragmentation *per se* tends to increase species diversity, but if the total amount of habitat is small, the situation is reversed. These debates highlight the ongoing controversy surrounding the independent effects of fragmentation *per se* on biodiversity. Moreover, the impacts of fragmentation are often species-specific (Haila, 1999), underscoring the need for further research, particularly on understudied biota such as bryophytes in specific ecosystems like fragmented subtropical forests, to better elucidate the mechanisms underlying habitat fragmentation’s effects on biodiversity.

The Chinese subtropical forest zone encompasses a vast area of approximately 2.5 million km², hosting high biodiversity. Prolonged intensive human disturbances and land-use led to forest loss at an alarming rate, leaving widely distributed secondary forests throughout this region. Currently, most Chinese subtropical forests were fragmented and in early- to mid-successional stages (Liu et al., 2016).

Bryophytes represent an integral component of subtropical forest ecosystems (Chen et al., 2009; Cai et al., 2017). As unique green land plants, they lack vascular tissues and exhibit a dominant gametophytic phase. Bryophytes have evolved a unique poikilohydric strategy, enabling them to absorb water across their entire surface through capillary action and to tolerate desiccation by entering a state of metabolic inactivity (Aranda et al., 2014). Furthermore, bryophytes are characterized by their small size, high habitat specificity, substrate selectivity, short generation time, and fast colonization-extinction rate (Pharo and Zartman, 2007; He et al., 2016). Consequently, the responses of bryophyte diversity to forest fragmentation *per se* in subtropical ecosystems are likely different from those observed in other biotas.

Forest fragmentation has long been recognized as a significant threat to biodiversity, with studies consistently demonstrating that forest loss has substantial negative impacts on species richness (SR) (Laurance et al., 2002), and genetic diversity (Aguilar et al., 2008). These deleterious effects are not confined to vascular plants; they also extend to bryophytes, as evidenced by numerous studies (Tangney et al., 1990; Berglund and Jonsson, 2001; Baldwin, 2004; Fritz et al., 2008; González-Mancebo et al., 2013; Patiño et al., 2014; Malombe et al., 2016; Müller et al., 2019). However, our understanding remains limited regarding the specific effects of fragmentation on bryophyte diversity within subtropical forest ecosystems. This research gap persists despite the ecological importance of bryophytes in these habitats.

Examining the effects of fragmentation *per se* on biodiversity, earlier researches have focused on patch (or island) characteristics such as number, mean size and isolation, following experimental or statistical control for habitat amount (Fahrig, 2003; Palmeirim et al., 2019). Besides these configuration-specific metrics relevant to habitat configuration, other variables, such as island shape irregularity, shape variability, size variability, and island number, also deserve consideration. These variables capture the heterogeneity of macro-environmental conditions within landscapes by describing the spatial arrangement of remaining habitats to varying degrees. Despite their potential relevance, these configuration-related variables have received disproportionately little attention in studies relevant to landscape fragmentation to date.

Land-bridge islands, particularly those formed by dam construction and subsequent inundation, provide an ideal experimental system for studying habitat fragmentation (Gotelli and Graves, 1990; Diamond, 2001; Terborgh et al., 2001; Wu et al., 2003; Terborgh and Feeley, 2008; Wang et al., 2010). These systems offer several advantages: all fragments originate simultaneously from a single, well-documented disturbance event, and their boundaries are clearly demarcated by water (Terborgh and Feeley, 2008). Unlike oceanic islands, dam-formed land-bridge islands generally occur over spatial scales comparable to terrestrial habitat patches and are relatively recent in origin. This temporal context minimizes confounding factors related to evolutionary adaptation, allowing for a more direct examination of fragmentation effects (Terborgh et al., 1997). Despite these methodological strengths, relatively few studies have been conducted to elucidate the impacts of fragmentation *per se* on biodiversity on land-bridge islands.

In ecological studies, understanding the impacts of habitat fragmentation *per se* on biodiversity remains a critical challenge. While previous research has explored the effects of fragmentation on various ecosystems, significant gaps remain in our understanding of its impacts on bryophyte assemblages. This study aims to address these gaps by examining the relative contributions of habitat fragmentation *per se* and its associated variables to bryophyte species richness (SR) and species composition (SC) in fragmented subtropical forests, thus to provide a scientific basis for the conservation of bryophyte diversity and new evidence to resolve the debate on the impact of habitat fragmentation *per se* on biodiversity.

## 2 Region and methods

### 2.1 Study region

**Figure 1:**
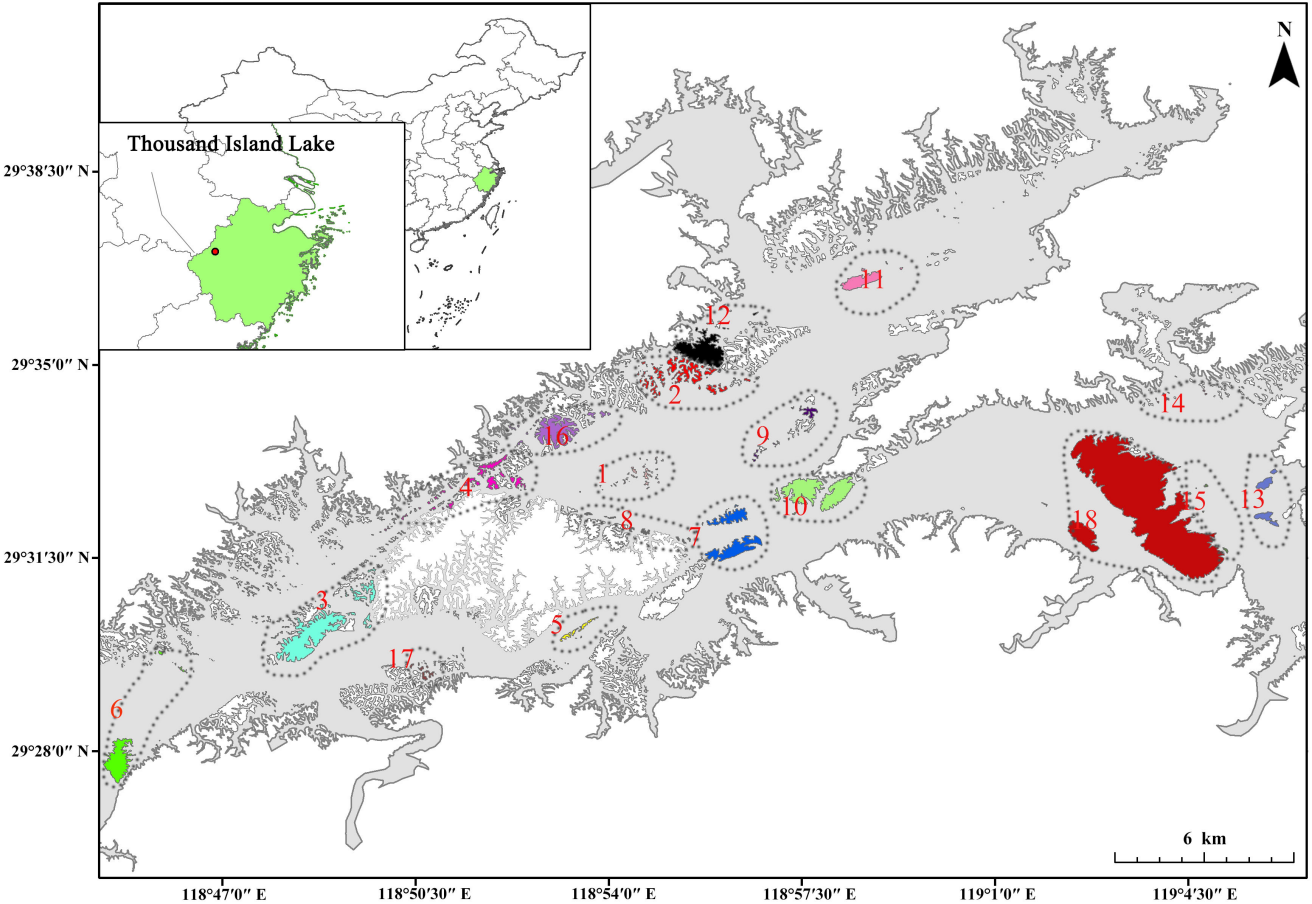
Map of island and landscape locations in the Thousand Island Lake region, Zhejiang Province, eastern China (18 landscapes with different colors, including 166 islands).

The Thousand Island Lake (TIL), located in Zhejiang province, China (29°22′-29°50′N and 118°34′-119°15′E), resulted from the damming of the Xin’an River in Chun’an County in 1959 (**Figure 1**). With the construction of the Xin’an River dam, an area of approximately 580 km^2^ was inundated, forming 1078 land-bridge forested islands (e.g. patches, 0.25–1320 ha) out of former hilltops when the water reached its final level (108 m) (Wang et al., 2009). The region exhibits a typical subtropical monsoon climate with pronounced seasonal variation, characterized by hot summers and cold winters. Mean annual temperature is 17.0°, ranging from 7.6° in January to 41.8° in July. Annual precipitation is 1430 mm (Wang et al., 2010).

During dam construction, primary forests in the region were extensively clear-cut, leading to near complete deforestation prior to lake inundation. Presently, most islands (formerly hilltops) are covered by secondary forests that regenerated naturally. These forests are dominated in the canopy by *Pinus massoniana*, with a sub-canopy and understory composed of a mix of broad-leaved trees and shrub species. The current vegetation structure has developed through subsequent secondary succession following the dam construction (Wilson et al., 2016). Since 1962, the region has been protected as a national park and has experience minimal human disturbances (Wilson et al., 2016).

The islands within the TIL are situated in a narrow geographic region characterized by similar climates, thereby minimizing the confounding impacts of different climate conditions on the relationships of bryophyte diversity with environmental variables associated with forest fragmentation. The TIL represents a highly fragmented subtropical forest landscapes with homogeneous matrix and synchronous vegetation successional history, providing an ideal setting to examine the impacts of fragmentation-related environmental variables on bryophyte assemblages in fragmented subtropical secondary forests.

### 2.2 Data collection

#### 2.2.1 Bryophyte inventory

This paper is part of a series of studies examining the bryophyte flora and biogeography of the TIL Archipelago. The preceding paper presented the sampling method for bryophyte inventory (Zhang et al., 2023). In the current study, we have analyzed 166 islands within the region, which exhibit significant variation in size, degree of isolation, shape, and perimeter (**Figure 1**).

The preliminary identification of family and genus was completed in the field. Voucher specimens of the bryophytes were collected, tagged, placed in kraft paper envelopes, and deposited at the Bryophyte Herbarium of Shanghai Normal University (SHTU). All specimens were identified in the laboratory by using a microscope. The nomenclature followed TROPICOS (Missouri Botanical Garden, 2022).

Previous studies have suggested that the number of sampling points per unit area tends to decrease with increasing island area (Gavish et al., 2012), which could introduce systematic biases in species sampling for larger islands. To address potential sampling biases associated with island size, we examined the relationship between sampling intensity and island area. To evaluate the adequacy of our sampling protocol for large islands, we implemented a rigorous analytical approach.

After randomizing the specimen collection sequence to minimize observer bias, we analyzed the relationships between cumulative species richness and cumulative sampling effort using an asymptotic function. This approach allowed us to estimate expected species numbers for large islands. We then calculated the sampling error (SE) as the relative difference between the expected (E) and observed (O) species richness ( $SE=\frac{E-O}{O}$ ×100%). A threshold of 10% was established to assess sampling adequacy. If the calculated SE exceeded this threshold for a given island, we conducted additional sampling to ensure robustness of our species estimates. This iterative process helped maintain the reliability of our data across the studied islands (Gotelli and Colwell, 2011).

#### 2.2.2 Environmental variables at the island level

Environmental data were initially collected at the island level. To get the area and perimeter data of the 166 islands, within the study region, we digitized all of them at a 1:5,000 scale employing SPOT-6 imagery, which boasts a high resolution of 1.5 meters for panchromatic and 6 meters for multispectral images (website: https://www.intelligence-airbusds.com/en/147-spot-6-7-satellite-imagery). The area and perimeter of the study islands were derived from the digitized maps using ArcGIS 9.3. These two variables were then used to generate the shape irregularity index (SHA). Shape irregularity reflects the relative proportion of edge habitat on an island, and it was calculated as follows (Patton, 1975):


$$SHA=\frac{P}{2\sqrt{A\pi }}$$

Where P is the perimeter in meters and A is the area (m^2^). The higher the SHA is, the more irregular the shape, with one representing a circular island.

The ecological isolation of islands in fragmented island systems is determined by multiple factors beyond just the distance to the mainland. Each island has its own local species pools of immigrants in fragmented island systems. The isolation of an island depends not just on the distance to the mainland (with a major species pool) but also on the area represented by the nearby islands (with their own local species pools). Namely, the degree of isolation for an island also depends on the amount of habitat within some distance of the island (Fahrig 2013). In the study region, the topographic and hydrographic features of the islands vary significantly (**Supplementary Figure S1**), and the shortest distance to the shore did not show substantial variation across these islands (**Figure 1**). It was thus inappropriate to take the shortest distance of an island to the shore as a measure of its isolation degree. To account for these factors, we adopted a modified measure of ecological isolation based on spatial analysis. We took the relative proportion of water within a circle of a diameter of 1000 m, which was centered on the study island, as the surrogate of its isolation degree (Berglund and Jonsson, 2001). Additionally, we recorded the maximum elevation of each study island by using a GPS device during the sampling process.

#### 2.2.3 Landscape-level environmental and species data

Based on the distribution, size, and configuration of the 166 islands, we identified 18 distinct regions, which roughly represented varying fragmental levels (**Figure 1**; **Supplementary Table S3**). For each region (comparable to a landscape unit), seven landscape-level environmental variables were derived from the ecological characteristics of the constituent islands (**Supplementary Table S3**). The isolation degree, mean area, and shape irregularity at the landscape level are averages of their corresponding values for all islands within the landscape. The total area is the sum of all island areas. Variability in island area and shape for each landscape was quantified as the standard deviation of island areas and shape irregularities across all islands in that landscape. To reduce the impact of multicollinearity on the results, in our analysis we included island number, total area, mean area, isolation degree, area variability, shape irregularity and shape variability, all of which were selected based on having a variance inflation factor (VIF) less than 10. A cube root transformation was applied to all environmental data prior to analysis.

The total area of a study landscape strongly influences habitat amount (Bird and Fahrig, 2013). Accordingly, island total area was selected as a proxy for habitat amount. The remaining six variables, which reflect varying aspects of habitat spatial configuration, treated as direct measures of habitat fragmentation *per se*. To disentangle the confounding effects of habitat amount loss and habitat fragmentation *per se* on bryophyte diversity, we incorporated island total area into relevant models as a covariate. Based on species data of each island, we obtained species data in each landscape region.

#### 2.2.4 Sampling for analyzing edge effects

The edge effects resulting from forest fragmentation may explain the influences of fragmentation on bryophyte assemblages. To investigate the potential edge effects of forest fragmentation on bryophyte assemblages, we selected thirteen study islands (**Supplementary Table S1**). In the selection process, we considered island shape, area, elevation, and isolation to maximize the range of selected conditions. Based on the size and topography of these islands, for each study island, we sampled two plots: an edge strip (10 m × 2 m) along the forest edge of the focal island (always located along the island’s edge) and a forest interior site (10 m × 10 m). To reflect forest interior environmental conditions, the distance from the center of the site to the edge of the forest (island) is not less than 15 m.

Five plots (2 m × 2 m) in each belt and 25 plots (2 m × 2 m) in each site were sampled. In each plot, a microcoenose sampling method was employed to investigate bryophyte coverage (Ilić et al., 2018). In this case, every bryophyte fragment was considered a microcoenose. Each plot was subdivided into four grids of the same size (1.0 m × 1.0 m), and a rigid frame (33 cm × 33 cm) was placed centrally within the largest bryophyte fragment in each grid. The rigid frame was partitioned into 100 squares (3.3 cm × 3.3 cm) using fine wires. The number of intersections where a species was present was counted to estimate its coverage within each grid (Guo and Cao, 2001). Coverage values were calculated for all grids, including those without bryophytes. Only the bryophyte species occurring on the soil, plant litter, tree bases, and roots (no more than 10 cm above the ground) were analyzed for species coverage. Additionally, epiphytic species in each plot were included in the study for SR analysis.

The species-area relationships for each belt and site were generated using PC-ORD v. 5.0 (McCune and Mefford, 1999), incorporating 95% confidence intervals and employing the first-order jackknife method (Palmer, 1990) to estimate their SR. For each site, the program randomly subsampled 25 plots, while belts were analyzed using five plots per replicate. The expected SR for each belt and site was determined through repeated subsampling according to the program’s default settings, thereby providing robust estimates of species richness.

### 2.3 Data analysis

#### 2.3.1 Contributions of landscape attributes in explaining variation bryophyte SR

The taxon-specific responses of SR to environmental variables have been well documented (Patiño et al., 2014). In our analysis, families were retained only if they occurred in more than one-third of the 166 islands. Ultimately, thirteen categories were included in our analyses: liverworts, Leucobryaceae, Leskeaceae, Pottiaceae, Fissidentaceae, Hypnaceae, Entodontaceae, Orthotrichaceae, Thuidiaceae, Brachytheciaceae, Bryaceae, Anomodontaceae, and Mniaceae (**Supplementary Table S4**).

Multivariate statistical methods including variance Partitioning, Redundancy Analysis, Canonical Correspondence Analysis) have been widely used to identify the independent explanatory roles of environmental variables on response variables (Capblancq and Forester, 2021; Smith and Lundholm, 2010; Baidya et al., 2025; Sadia et al., 2025; He et al., 2024). In this study, we employ these approaches to quantify the relative contributions of total habitat (using area as its proxy) and habitat fragmentation processes to bryophyte SR and SC. The response variables encompassed SR values for the fourteen categories across the 18 landscapes (**Supplementary Table S4**), while the explanatory variables included the environmental variables of these landscapes (**Supplementary Table S3**). Redundancy Analysis (RDA) was performed, as the first ordination axis from DCA (Detrended Correspondence Analysis) exhibited a length of 0.8 standard deviations (Lepš and Šmilauer, 2003; ter Braak and Šmilauer, 2012).

A Monte Carlo permutation test based on 999 random permutations was conducted to evaluate the significance of the eigenvalues of all canonical axes. Variance Partitioning was employed to decompose the variation in the response data into components explained by the focal explanatory variable alone and the confounded variation between the focal variable and the remaining covariates (Borcard et al., 1992). After centering the response variables (mandatory for the RDA), we set other parameters to their default values in the software.

#### 2.3.2 Contributions of landscape attributes in explaining the variation of SC

The canonical correspondence analysis (CCA) was applied (Lepš and Šmilauer, 2003; ter Braak and Šmilauer, 2012) to assess the relative contributions of landscape attributes in explaining the variation of bryophyte SC. Species presence/absence data (**Supplementary Table S2**) were used as response variables, while the environmental variables of the 18 landscapes (**Supplementary Table S3**) served as explanatory variables. During analysis, rare species were excluded from weighting, and all other parameters were set to their default values in the software.

We performed the RDA, CCA, Monte Carlo permutation tests, and Variation Partitioning using CANOCO for Windows 5.0 (ter Braak and Šmilauer, 2012). Using the vegan 2.5-4 package in R, we quantified the regression relationships SR with landscape attributes, with landscape attributes serving as the sole constraining variable.

#### 2.3.3 Comparison of bryophyte assemblages between edge and interior environments

Each study in interior site included 25 plots, where the corresponding edge contained only five plots. Bryophyte coverages and species number of five plots for each interior environments site were recorded as the mean values of 1000 random five-plot samples without replacements (i.e., the same plot number as the corresponding edge). The data presented are the mean ± standard errors (SEs). Analysis of variance (ANOVA) was used to test the differences between edge and interior environments site in bryophyte SR and coverage using the procedures in the SPSS 22.0 statistical package (SPSS Corp.). Expected SR comparisons were also conducted.

## 3 Results

### 3.1 General sampling aspects

The study 166 islands highly varied in island area (0.037 - 869.035 ha), perimeter (72 - 32019 m), maximum elevation (94 - 379 m), degree of isolation (0.034 - 0.993), and shape irregularity (1.059 - 4.589) (**Supplementary Figure S1**; **Supplementary Table S1**). The 18 landscapes also varied in island number (2 - 29), total area (3.129 - 870.295 ha), total perimeter (1642.2 - 32625.9 m), mean area (0.447 - 435.147 ha), mean maximum elevation (94.7 - 271.0 m), area variability (0.669 - 3.301), shape irregularity (1.207 - 2.528), shape variability (0.078 – 0.534), and isolation degree (0.248 - 0.97) (**Figure 1**; **Supplementary Table S3**).

A total of 189 bryophyte species were identified based on 5,169 specimens collected across the 166 islands, including 25 liverwort species (21 genera and 16 families) and 164 moss species (85 genera and 34 families). The most species-rich families were Brachytheciaeae (18 species, 9.52%), Pottiaceae (19, 10.05%), Bryaceae (13, 6.88%), and Hypnaceae (13, 6.88%). The genera with the highest SR were *Bryum* (12 species, 6.35%), *Brachythecium* (10, 5.29%), *Entodon* (10, 5.29%), and *Fissidens* (9, 4.76%). Pleurocarpous mosses (85 species, 44.97%) slightly outnumbered acrocarpous mosses (79, 41.80%). Bryophyte SR exhibited a significant variation across the 18 regions (landscapes), ranging from 26 to 121 species (**Supplementary Table S2**).

### 3.2 Contribution of fragmentation *per se* to bryophyte SR

The result of the RDA indicate that the explanatory variables significantly accounted for 59.5% of the total variation in bryophyte SR across the 18 landscapes (permutation test: all axes, pseudo‐*F* = 2.1, *P* = 0.07; first‐axis, pseudo‐*F* = 9.8, *P* = 0.08), leaving 40.5% unexplained. This substantial explanatory power indicates that the nine environmental variables significantly influenced bryophyte SR variation among the landscapes (**Supplementary Table S5**).

If involved in the analysis as the only constraint variable, island shape irregularity (27.5%, *P* = 0.01) and total area (20.5%, *P* = 0.038) were the primary predictors significantly influencing SR. The shape variability (14.3%, *P* = 0.092), isolation degree (13.3%, *P* = 0.082), mean area (12.1%, *P* = 0.112), island number (11.0%, *P* = 0.166), and area variability (7.0%, *P* = 0.270) all exerted influences on bryophyte SR to varying degrees (**Table 1**).

**Table 1:** Percentage variation in species richness (SR) of 14 bryophyte categories among 18 landscape regions explained by environmental variables in RDA estimated using two different methods.

Landscape-level variables	Simple effects			Conditional effects		
	% of explained variation	pseudo-F	*P*-values	% of explained variation	pseudo-F	*P*-values
Shape irregularity	27.5	6.1	0.01	27.5	6.1	0.009
Total area	20.5	4.1	0.038	3	0.7	0.469
Shape variability	14.3	2.7	0.092	5.3	1.3	0.265
Isolation degree	13.3	2.5	0.082	3.1	0.8	0.467
Mean area	12.1	2.2	0.112	3.6	0.9	0.374
Island number	11	2	0.166	14.4	3.7	0.029
Area variability	7	1.2	0.27	2.5	0.6	0.55

Simple effects: percentage variance explained by an individual variable while used as the only constraining variable. Conditional effect: additional variance explained by the variable at the time it was included in the stepwise selection. Displayed were percentages of explained variation and contribution, and with values of pseudo-F statistics and *p*-values.

Based on the forward selection analysis, island shape irregularity (20.5% of explained variation, *P* = 0.038) imposed the strongest influences on bryophyte SR, followed by island number (14.4%, *P* = 0.029). Other five variables accounted for 2.5% to 5.3% of the explained variation (**Table 1**).

Variance partitioning revealed that fragmentation *per se* and island total area (habitat amount) independently explained 38.92% (*P* = 0.173) and 6.2% (*P* = 0.213) of the variation in bryophyte SR, respectively. Among the six environmental variables associated with fragmentation *per se*, island number (16.2%, *P* = 0.034) and shape irregularity (15.5%, *P* = 0.041) exerted most strong effects on SR, followed by island mean area (8.5%, *P* = 0.130), shape variability (5.3%, *P* = 0.239) and isolation (4.3%, *P* = 0.303), while area variability exhibited insignificant effects on SR (**Table 2**).

**Table 2:** Variance partitioning resulting from the partial RDA of environmental variables on bryophyte SR.

Landscape-level variables	Variation	% of explained variation	% of all variance	DF	F	*P*-value
Island number	0.1619/0.0000	27.2	16.2	1	4	0.034
Mean area (MA)	0.0847/0.0359	14.2	8.5	1	2.1	0.13
Total area (TA)	0.0617/0.1436	10.4	6.2	1	1.5	0.213
Area variability (VA)	0.0207/0.0496	3.5	2.1	1	0.5	0.641
Shape irregularity	0.1545/0.1206	26	15.5	1	3.8	0.041
Shape variability	0.0533/0.0898	9	5.3	1	1.3	0.239
Isolation degree	0.0425/0.0907	7.2	4.3	1	1	0.303
Habitat fragmentation *per se*	0.3892/0.1436	65.41	38.92	5	1.5	0.173

Variation effect of the focal variable/confounded effect of the focal environmental variable with the others; DF, F and P-value: parameters for the focal variable in the same row. Habitat fragmentation *per se* including all variables except island total island.

**Table 1** and **Table 2** demonstrate that the influence of fragmentation *per se* on bryophyte SR is stronger than that of habitat amount, as measured by total area. Furthermore, the effect of island number and shape irregularity on bryophyte SR are more pronounced than those of the other four variables associated with fragmentation *per se* investigated.

**Figure 2:**
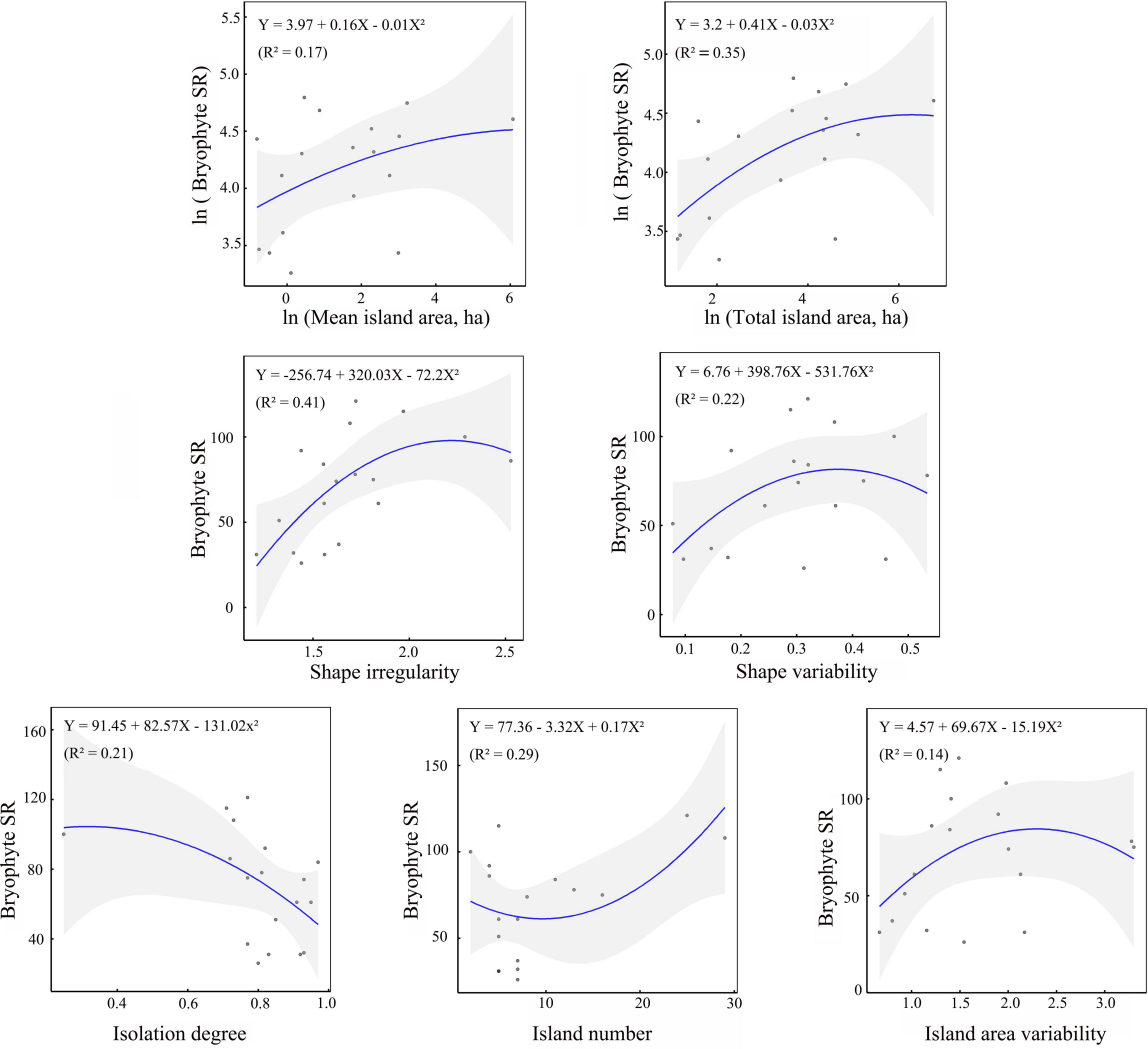
Relationships of bryophyte SR with seven environmental attributes (isolation degree, island number, island area variability, mean and total island area, shape irregularity and shape variability). Solid lines represent model fit and shaded areas represent 95% confidence intervals.

The effects of seven environmental variables on bryophyte SR exhibited distinct patterns. As the only constraining variable, island number, shape irregularity, mean area and total area all exerted a positive linear effect on bryophyte SR, and isolation degree exerted a negative linear effect. Interestingly, the variabilities in both island area and shape exhibited a nonlinear relationship with bryophyte SR, following a unimodal model (**Figure 2**). These patterns were consistent across liverworts, mosses, and both acrocarpous and pleurocarpous mosses (**Supplementary Figure S2**).

### 3.3 Contribution of fragmentation *per se* to bryophyte SC

The result of the CCA indicate that the explanatory variables significantly explained 45.9% of the total variation in bryophyte SC across 18 landscapes (permutation tests: all axes, pseudo‐*F* = 1.2, *P* = 0.004; first‐axis pseudo‐*F* = 1.2, *P* = 0.01) (**Supplementary Table S6**).

As the only constraining variable in the analysis, total area (9.4% of explained variation, *P* = 0.006), mean area (9.8%, *P* = 0.007), isolation degree (9.8%, *P* = 0.007) were identified as the primary constraints of bryophyte SC in the study region. Island shape irregularity (7.3%, *P* = 0.052), shape variability (6.6%, *P* = 0.11), and island number (6.6%, *P* = 0.178) had comparable influences on bryophyte SC (**Table 3**).

**Table 3:** Percentage variation in bryophyte SC among 18 landscape patches of the Thousand Island Lake (TIL) explained by environmental variables in CCA estimated using two different methods.

Landscape-level variables	Simple effects			Conditional effects		
	% of explained variation	pseudo-F	*P*-values	% of explained variation	pseudo-F	*P*-values
Mean area	9.8	1.7	0.002	6.1	1.1	0.292
Isolation degree	9.8	1.7	0.012	7.4	1.3	0.02
Total area	9.4	1.7	0.002	9.4	1.7	0.002
Shape irregularity	7.3	1.3	0.052	6	1.1	0.33
Island number	6.6	1.1	0.178	5.9	1.1	0.324
Shape variability	6.6	1.1	0.11	5.4	1	0.576
Area variability	5.3	0.9	0.734	5.7	1	0.43

Simple effects: percentage variance explained by an individual variable while used as the only constraining variable. Conditional effect: additional variance explained by the variable at the time it was included in the stepwise selection. Displayed were percentages of explained variation and contribution, and with values of pseudo-F statistics and *P*-values.

The forward selection analysis revealed that total area and isolation degree imposed significant effects on bryophyte SC, accounting for 9.4% (*P* = 0.002) and 7.4% (*P* = 0.02) of the explained variation. Island mean area, shaper irregularity, island number exerted weak effects on bryophyte SC, accounting for 5.9% to 6.1% of the explained variation.

Variance partitioning revealed that fragmentation *per se* and total area independently accounted for 36.5% (*P* = 0.042) and 5.9% (*P* = 0.362) of the total variation in bryophyte SC across the landscapes, respectively. Additionally, All the six variables associated with fragmentation *per se* exerted comparable effects on bryophyte SC, accounting for 5.6% to 6.6% of the total variation (**Table 4**).

**Table 4:** Variance partitioning resulting from the partial CCA of environmental and partial variables on bryophyte species composition (SC).

Landscape-level variables	Variation	% of explained variation	% of all variation	DF	F	*P*-value
Island number	0.0821/0.0098	12.9	5.9	1	1.1	0.328
Mean area	0.0781/0.0582	12.3	5.6	1	1	0.406
Total area	0.0819/0.0478	12.9	5.9	1	1.1	0.362
Area variability	0.0896/0.0000	14.1	6.5	1	1.2	0.218
Shape irregularity	0.0916/0.0092	14.4	6.6	1	1.2	0.226
Shape variability	0.0877/0.0035	13.8	6.3	1	1.2	0.262
Isolation degree	0.0852/0.0499	13.4	6.1	1	1.1	0.302
Habitat fragmentation *per se*	0.5060/0.0479	79.6	36.5	6	1.1	0.042

Variation effect of the focal variable/confounded effect of the focal environmental variable with the others; DF, F and P: parameters for the focal variable *per se* in the same row. Habitat fragmentation *per se* including all variables except island total island.

### 3.4 Edge effects on bryophyte assemblages

**Figure 3:**
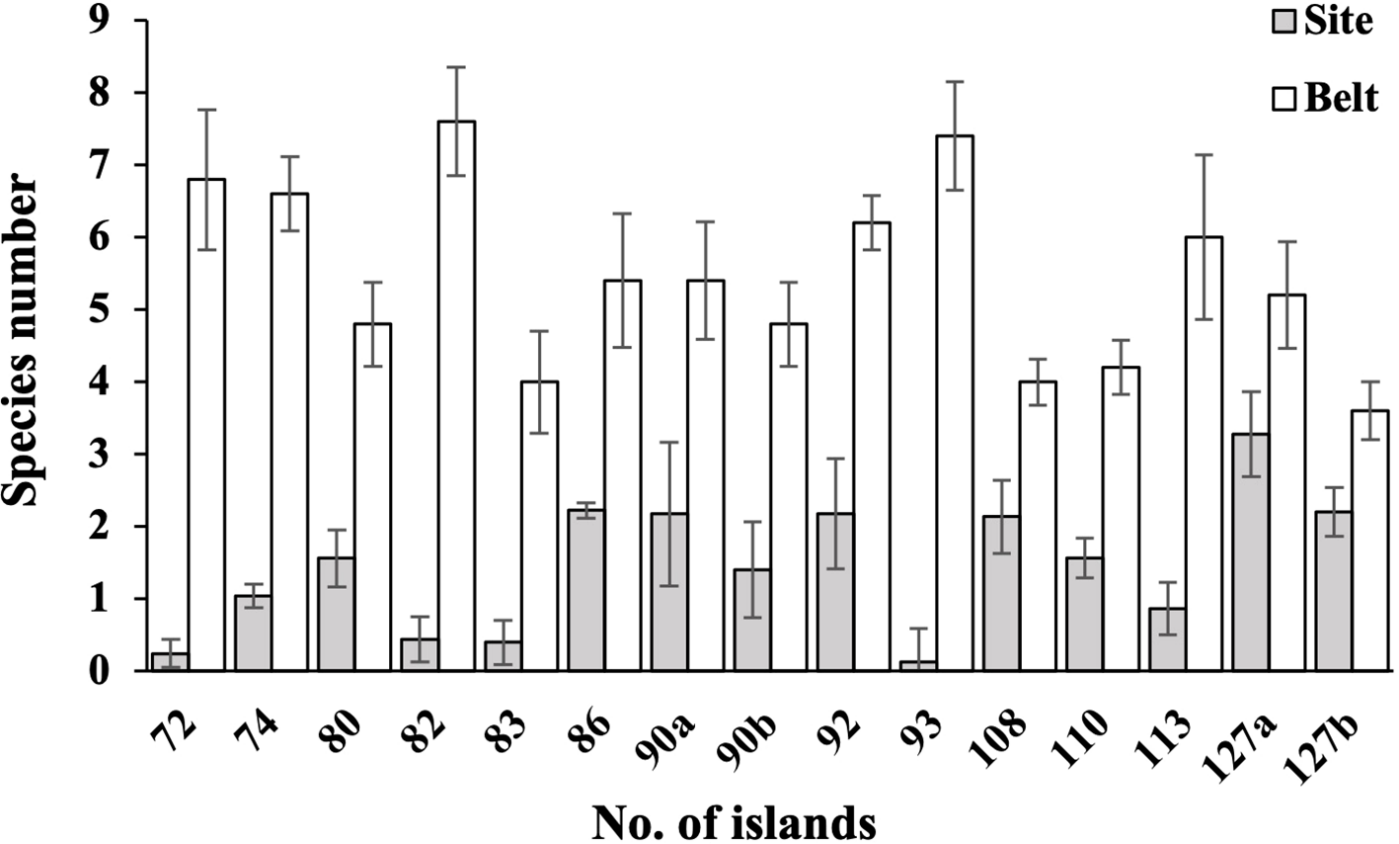
Species number of bryophytes in 15 belts on forest edges and 15 sites in the interior of 13 forest islands. The coverage and species number of bryophytes in belts are significantly higher than those of sites (all *P*< 0.01).

**Figure 4:**
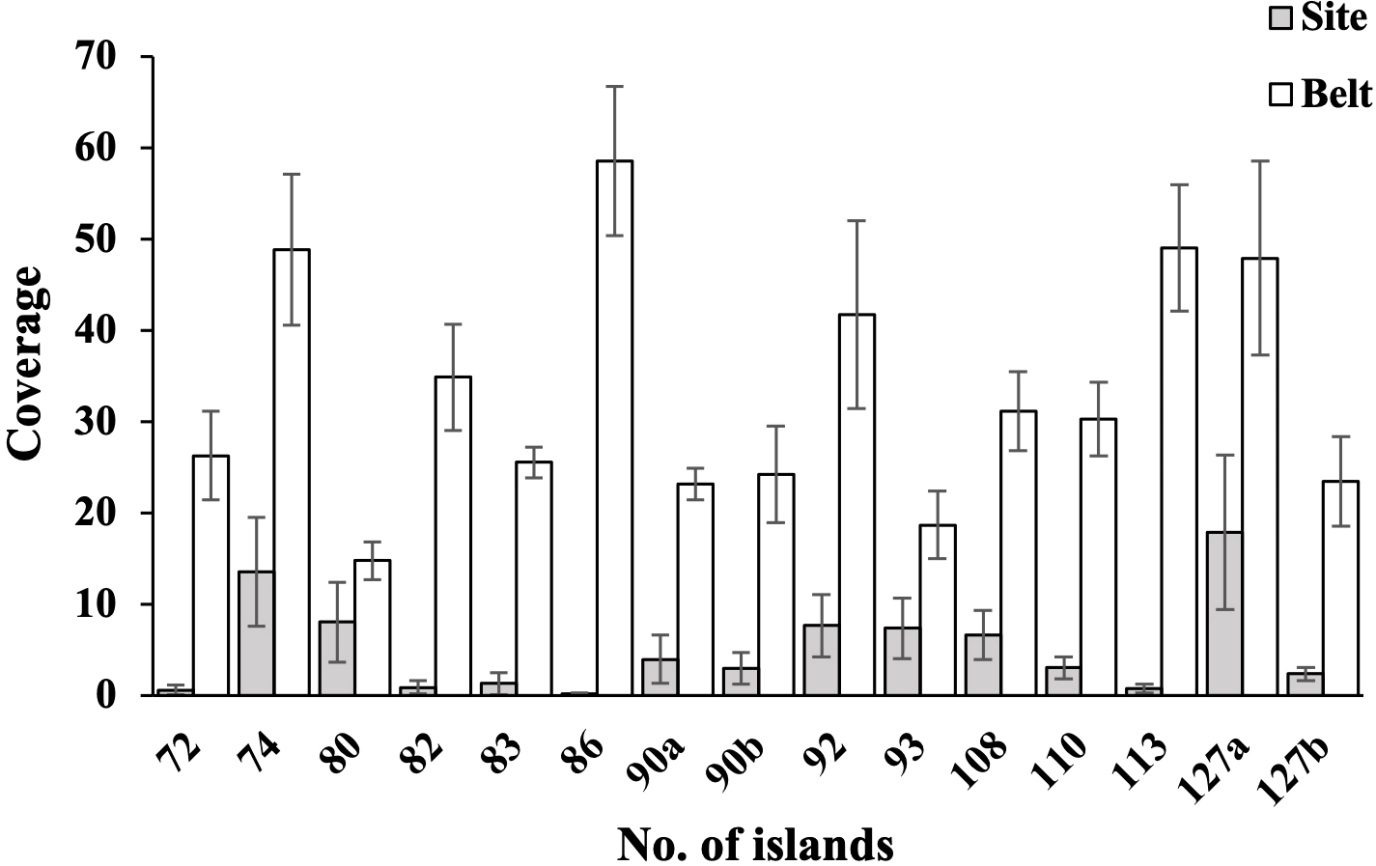
Coverage of bryophytes in 15 belts on forest edges and 15 sites in the interior of 13 forest islands. The coverage and species number of bryophytes in belts are significantly higher than those of sites (all *P*< 0.01).

The bryophyte coverages and SR in the 13 forest islands exhibited significantly higher values in edge zones compared to interior sites, with statistical significance at *P<* 0.01 (**Figures 3**, **4**). This pattern demonstrates a pronounced positive edge effects on bryophytes assemblages in the study region. Litter coverage measurements on the forest floors revealed significant differences between interior sites and edge belts. Specifically, litter coverage in plots for interior sites ranged from 25% to 100% (mean = 88.5%), while belts exhibited lower values, ranging from 0% to 80% (mean = 41.1%). In contrast, canopy coverages in plots showed a marked reversed pattern between the two zones, with sites ranging from 65% to 90% (mean = 85.0%) and belts ranging from 0% to 60% (mean = 45.0%).

## 4 Discussion

This study represents the first comprehensive quantification of the relative contributions of habitat fragmentation *per se* and its associated environmental variables to bryophyte SR and SC in fragmented subtropical secondary forests at the landscape scale within a single evaluation. Our analyses revealed that habitat fragmentation *per se* exerted strong and significant effects on bryophyte assemblages at the landscape scale, a finding consistent with Fahrig (2017) conclusion that most biodiversity responses to habitat fragmentation *per se* were positive, regardless of whether fragmentation is measured as patch number, edge length, mean patch size, or as a whole-landscape metric. Among the six landscape attributes associated with fragmentation *per se*, island number, mean area and shape irregularity all demonstrated linear and positive effects on bryophyte SR to varying extents, while shape variability and area variability exhibited a nonlinear relationship with bryophyte SR, basically followed a unimodal model, indicating moderate variabilities of island shape and area were more conducive to maintain bryophyte diversity than weaker or stronger levels of variability. Importantly, the effects of habitat fragmentation *per se* on bryophyte SR and SC were significantly stronger than those of habitat amount, as represented by total island area, in the studied fragmented subtropical secondary forest landscape.

### 4.1 Positive edge effects of fragmented forest patches on bryophyte SR

Dense grasses and forests are not conducive to the growth of most bryophytes, most bryophytes prefer to grow in open, sparse forests (Guo and Cao, 2001; Jiang et al., 2018; Löbel et al., 2006). For example, Vellak et al. (2003) highlighted that the tree layer, particularly the proximity to the nearest tree, was a key factor influencing ground bryophyte diversity. Fergus et al. (2017) found that an increase in vascular plant SR and canopy cover led to a corresponding decrease in bryophyte SR. In dry grasslands on the Baltic island of Öland, Sweden, Löbel et al. (2006) observed a negative correlation between bryophyte SR and vascular plant cover. In a subtropical forest, Jiang et al. (2018) conducted a study across a forest edge-to-interior gradient and found that bryophyte richness increased along an interior-to-edge gradient, which was due to the reduced bryophyte SR under closed canopies. In the study region, the edge zones of fragmented forests were characterized by high light intensity, low humidity, open habitats and empty niches, which is beneficial to bryophytes. Our findings confirmed strong and positive edge effects on bryophyte SR and coverage in fragmented subtropical secondary forests (**Figure 3**). The positive edge effects provide a plausible explanation for the observed relationships between bryophyte SR and ecological variables including island number, shape irregularity and its variability, and island area variability.

### 4.2 Island total area, mean area and area variability

Understanding the effects of fragmentation *per se* on biodiversity requires careful selection of metrics to quantify fragmentation. Some environmental variables directly relate to habitat loss (e.g., island total area) or changes in the spatial configuration (shape), while others indirectly affect spatial configuration (e.g., island number, shape and area variability) (Didham et al., 2012). Including indirect variables to assess the impacts of fragmentation on biodiversity has been shown to enhance understanding of the mechanisms involved (Wilson et al., 2016). In the study region, the landscape-level area variability, and shape variability indirectly influences the spatial configuration of fragmented patches, which is consistent with the conclusion by Didham et al. (2012) to an extent.

Species richness increasing with area is one of the most fundamental ‘laws’ in ecology (Schoener, 1976; Lawton, 1999; Lomolino, 2000; Loke et al., 2019). Fragmentation (*s.l.*), which reduces the total area, has consistently been associated with negative impacts on biodiversity (Fahrig, 2003; Fahrig, 2013). In our study region, we observed a positive relationship between total island area and bryophyte species richness, though the significance of this effect was relatively low (**Table 1**). The lesser influence of total area on bryophyte SR at the landscape level is likely due to its relatively smaller variability across landscapes compared to other five environmental variables.

In the subtropical fragmented forests of the TIL, Landscape-level island mean area exerted linear and positive impacts on bryophyte SR in logarithmic form. Due to the minimum patch size requirement for each species (Díaz et al., 2000), small islands with limited habitats struggled to maintain larger populations, which were more prone to stochastic extinction. However, Riva and Fahrig (2023) reported the opposite conclusion, they found that species richness actually decrease with increasing mean patch size across sets of patches. The relationships between environmental variable and biodiversity outcomes is not straightforward, varying based on factors like geographic location, species ecological and physiological characteristics, and environmental conditions (Zhang et al., 2024). For a fragmented landscapes with a fixed total area, the number of large islands would become scarce if the island mean area within the landscape is large. Often large islands have diverse and specialized habitats, then the species that need to be distributed in special habitats would be unlikely to survive in landscapes that lack large islands

The landscape-level variability of island area indirectly influences the spatial configuration of fragmented patches (Didham et al., 2012). Within the study region, bryophyte SR exhibited a unimodal response to island area variability, as shown in **Figure 2**. In fragmented subtropical forest landscapes, increasing island area variability likely enhanced landscape heterogeneity to a certain extent, leading to an increase in bryophyte SR. However, once island area variability exceeds a critical threshold, the landscape may contain a mix of extremely large and (or) very small islands, which would negatively impact bryophyte SR, as demonstrated in **Figure 2**. This phenomenon can be attributed to the following reasons.

Geometrically speaking, large patches have a lower edge-to-interior environment ratio than small patches if they have similar shapes. Consequently, bryophyte SR would decrease if a landscape includes some very large forest fragments. This is because the positive edge effects on bryophyte SR in large forest fragments would diminish.

While for very small islands, they often lack sufficient habitats to sustain species populations, as each species requires a minimum patch size for habitation (Díaz et al., 2000). At the population level, insufficient habitat areas cannot support larger populations, which are more prone to stochastic extinction (MacArthur and Wilson, 1967). Therefore, bryophyte SR would also decrease if a landscape includes some very small fragmented patches.

### 4.3 Shape irregularity and shape variability

Our results revealed that landscape-level island shape irregularity show a positive and linear relationship with bryophyte SR. This phenomenon is straightforward to explain. It is because, under a given area, a more irregular island shape results in a larger perimeter, thus lead to a higher proportion of forest edge habitats, thereby fostering higher bryophyte abundance through the positive edge effect as stated above. Our results are consistent with those of Gignac and Dale (2005), showing that bryophyte SR is positively associated with shape irregularity in the low-boreal forest of Western Canada.

In comparison, shape variability exhibits a nonlinear relationship, following a unimodal model as illustrated in **Figure 2**. With an increase of shape variability across islands within a landscape, the landscape-level heterogeneity of macro environments likely increases to a certain extent. When such variability exceeded a certain threshold, the landscape would feature both relatively regular and highly irregular islands. For an island with a relatively regular shape (e.g., **Supplementary Figure S3-A**), the positive edge effects on bryophyte SR would diminish or become negligible due to the low edge ratio to the interior environment of the island. In the study region, many islands are characterized by multi-branched or strip-branched ridges (**Supplementary Figures S3-B-D**). Consequently, islands, particularly for small islands, if with very irregular shapes, would often exhibit long and narrow ridges (**Supplementary Figure S3-D**), creating more arid habitats that would reduce bryophyte SR. Consequently, islands with particularly irregular shapes are also unfavorable for the distribution of bryophytes.

### 4.4 Island number

At the landscape scale, island number is one of the important variables related to habitat fragmentation *per se* when controlling for changes in the total area (Fahrig, 2003). Fragmentation degree increases with increasing island number of a landscape for a given area size. Variance partitioning revealed that island number had a significant impact on bryophyte SR at the landscape level (**Table 2**).

Interestingly, at the landscape scale, bryophyte SR exhibit a nonlinear relationship with island number, with bryophyte SR generally being lower in landscapes containing moderate numbers of islands, and higher in those with either exceptionally small or exceptionally large island configurations. Generally speaking, islands tend to be larger when there are few islands at a give landscape scale, which tends to facilitate the distribution of taxa requiring special habitats in large island, thereby increasing bryophyte SR. In contrast, islands are more likely to be smaller when there are many islands at a given landscape scale, which may accentuate the edge effect and favor the distribution of bryophytes. From the perspective of the SLOSS strategy, the former scenario favors the SL strategy for diversity conservation, while the latter favors the SS strategy. Sun et al. (2008) reported marked differences in woody species richness across various island combination with islands of varying sizes in the TIL. With a fixed total area, they found that mixing islands of different sizes exhibited the highest woody SR, followed by the combination with smaller islands. In contrast, large islands supported the lowest levels of species richness (SR). Their findings partially align with our results on the impact of island number and area variability on biodiversity, except that woody plants exhibited a distinct response pattern compared to bryophytes.

### 4.5 Isolation

Dispersal limitations in island ecosystems have been extensively studied, particularly in relation to their ability to colonize distant sites. Bryophytes are often considered as long-distance dispersers by spores. Sundberg et al. (2006) demonstrated that the effect of isolation did not apply to bryophytes on islands isolated by a distance shorter than 40 km. Patiño et al. (2013) found that geographical isolation didn’t influence bryophyte SR on islands. From a theoretical perspective, bryophytes should have had large ranges because of their long-distance dispersal capacities. However, only a few species are really ubiquitous, and many species are restricted to specific regions (Frahm, 2008). Despite evidence of prolific spore production in some bryophyte taxa (Longton, 1997), and long-distance dispersal observed in some bryophytes (Vanderpoorten et al., 2008), many bryophytes are considered dispersal-limited at local scales (Haila, 1999; Hedenås et al., 2003; Chen et al., 2015). Habitat specialists of bryophytes exhibit limited dispersal capacities (Pimm et al., 2014); most experimental findings consistently show that major diaspores deposited within centimeters of the parent sporophytes (Miles and Longton, 1992). Establishment experiments and studies of patch-occupancy distributions demonstrate that dispersal limitation could explain species absences in fragmented habitats (Pharo and Zartman, 2007). Although some bryophytes are capable of long-distance dispersal, this ability is affected by other environmental factors. For example, long-distance dispersal often happens at higher elevations, but diaspores of the species on forest floors are often unable to get into these altitudes (Frahm, 2008). Additionally, ecological boundaries and topographical features can impose further dispersal constraints on some bryophytes. Therefore, the effects of island isolation on bryophyte assemblages to varying degrees have been reported (Tangney et al., 1990; Yu et al., 2019). At the island scale, isolation effect on bryophyte assemblages was also detected for the bryophytes in the study region (Zhang et al., 2023). Our findings suggest that habitat isolation affects bryophyte diversity patterns not only at the island scale but also at the landscape scale.

## 5 Conclusions

In fragmented subtropical secondary forests, the effect of habitat fragmentation *per se* on bryophyte SR and SC was significant, much stronger than that of habitat loss. Mean area and shape irregularity exhibited varying positive linear relationships with bryophyte SR. Isolation not only affected bryophyte SC, but also has a negative impact on SR. Island number, shape variability and area variability all displayed a nonlinear relationship with bryophyte SR, following a unimodal model. Quantifying the impacts of landscape fragmentation attributes on bryophyte SR and SC is crucial for the conservation of their diversity in fragmented subtropical secondary forests. Our findings suggest that, in such fragmented environments, the optimal strategy for bryophyte conservation within a given geographic range is to establish reserves in regions characterized by numerous forest patches with irregular shapes, large total area, and moderate variation in island shape and area. To effectively conserve bryophyte diversity, different SLOSS strategies should be considered, tailored to the specific characteristics of landscape fragmentation.

## Data Availability

The original contributions presented in the study are included in the article/**Supplementary Material**. Further inquiries can be directed to the corresponding authors.

## Supplementary material

Supplementary Figure 1 A region with islands of multi-long and irregularly branched topography in the TIL.

Supplementary Figure 2 Relationships of SR with seven environmental variables for four bryophyte categories. Environmental variables include isolation degree, island number, island area variability, mean and total island area, shape irregularity and shape variability. Four bryophyte categories include acrocarpous mosses, pleurocarpous mosses, moss and liverworts. Solid lines represent model fit and shaded areas represent 95% confidence intervals.

Supplementary Figure 3 Four examples showing variability of island shape in the TIL.

Supplementary Table 1 Environmental variables in 166 islands in the Thousand Island Lake (Zhang et al., 2023). The number with an asterisks indicates that one belt on the edge and a corresponding site in the interior was sampled on the island.

Supplementary Table 2 Occurrences of 189 species in 18 fragmented forest landscapes in the TIL.

Supplementary Table 3 Environmental information of 18 fragmented forest landscapes in the Thousand Island Lake (TIL). Landscape 1-18 were marked in **Figure 1**.

Supplementary Table 4 Species number of 14 bryophyte categories in 18 fragmented forest landscapes in the Thousand Island Lake (TIL).

Supplementary Table 5 Results of redundancy analysis on relationships of bryophyte SR with environmental variables in the TIL, summarizing eigenvalues, explained variation and additional statistics for each of the four ordination axes. Total variation is 1363.0000, explanatory variables account for 53.5% (adjusted explained variation is 31.1%. Test of significance of first canonical axis: pseudo-F = 9.8, P = 0.082. Test of significance of all canonical axes: pseudo-F = 21, P = 0.072.

Supplementary Table 6 Results of canonical correspondence analysis on relationships between bryophyte SC and environmental variables in the TIL, summarizing eigenvalues, explained variation and additional statistics for each of the four ordination axes. Total variation is 1.3851, explanatory variables account for 45.9% (adjusted explained variation is 8.0%. Test of significance of first canonical axis: pseudo-F = 1.2, P = 0.01. Test of significance of all canonical axes: pseudo-F = 1.1, P = 0.004.
