# Supplementary figures and images for: Positive effects of forest fragmentation per se on bryophyte diversity in subtropical fragmented forests: evidence from land-bridge islands
Source: Front Plant Sci. 2025 Apr 10;16:1539513. doi: 10.3389/fpls.2025.1539513 (PMC12018535; doi:10.3389/fpls.2025.1539513)

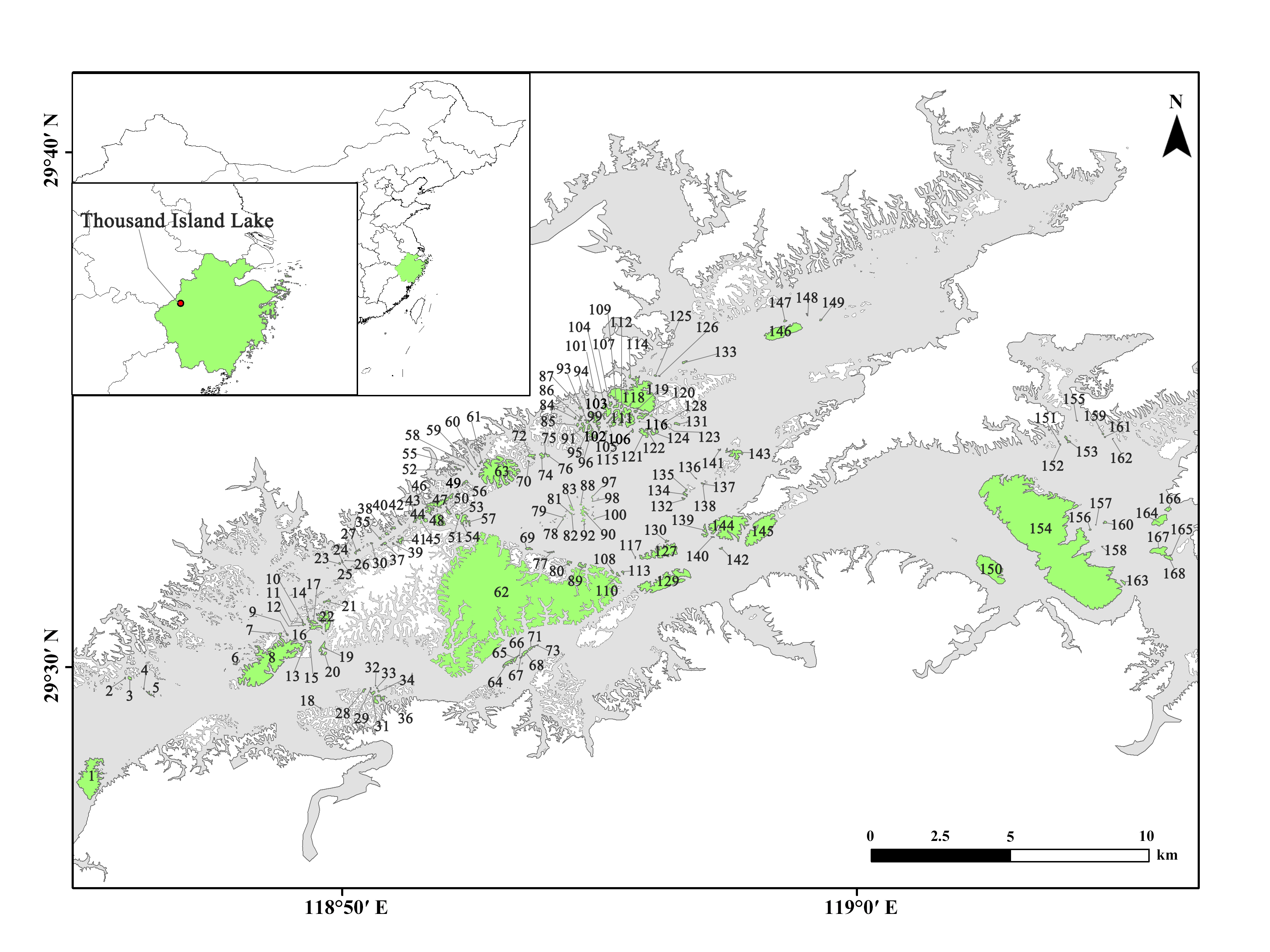

Supplement: Supplementary Figure 1 — A region with islands of multi-long and irregularly branched topography in the TIL. [file Image1.jpeg]

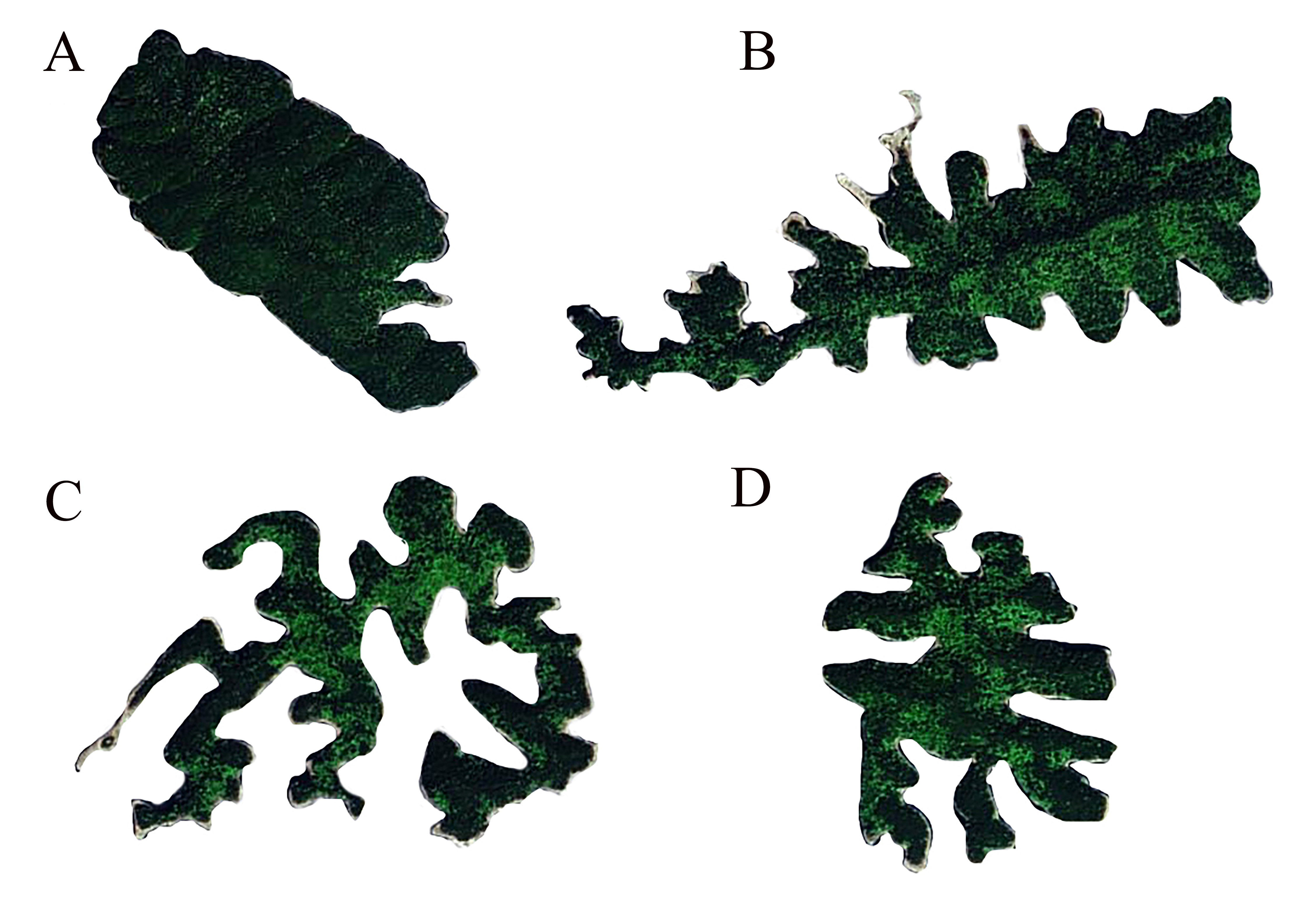

Supplement: Supplementary Figure 3 — Four examples showing variability of island shape in the TIL. [file Image3.jpeg]
