# Supplementary material for: Positive effects of forest fragmentation per se on bryophyte diversity in subtropical fragmented forests: evidence from land-bridge islands
Source: Front Plant Sci. 2025 Apr 10;16:1539513. doi: 10.3389/fpls.2025.1539513 (PMC12018535; doi:10.3389/fpls.2025.1539513)
Supplement: Supplementary Table 3 — Environmental information of 18 fragmented forest landscapes in the Thousand Island Lake (TIL). Landscape 1-18 were marked in Figure 1 . [file Table3.docx]

Table S3. Environmental information of 18 fragmented forest landscapes in the Thousand Island Lake (TIL)

| No. of Landscape | Lon (º) | Lat (º) | Island number | Total island perimeter (m) | Total island area (ha) | Mean island area (ha) | Island area variability | Shape irregularity | Shape variability | Mean maximum elevation (m) | Isolation degree |
| --- | --- | --- | --- | --- | --- | --- | --- | --- | --- | --- | --- |
| 1 | 118.910 | 29.550 | 11 | 4024.5 | 4.917 | 0.447 | 1.398 | 1.555 | 0.321 | 100.5 | 0.970 |
| 2 | 118.921 | 29.580 | 29 | 26570.0 | 69.497 | 2.396 | 1.980 | 1.693 | 0.368 | 106.1 | 0.730 |
| 3 | 118.821 | 29.511 | 16 | 28441.0 | 165.513 | 10.345 | 3.301 | 1.814 | 0.420 | 113.0 | 0.770 |
| 4 | 118.854 | 29.544 | 25 | 18674.8 | 39.800 | 1.592 | 1.489 | 1.723 | 0.320 | 103.2 | 0.770 |
| 5 | 118.892 | 29.504 | 7 | 3428.5 | 6.084 | 0.869 | 1.030 | 1.558 | 0.243 | 100.0 | 0.950 |
| 6 | 118.767 | 29.494 | 5 | 10058.7 | 79.154 | 15.831 | 2.130 | 1.840 | 0.370 | 121.0 | 0.910 |
| 7 | 118.935 | 29.535 | 4 | 14928.8 | 81.963 | 20.491 | 1.209 | 2.528 | 0.295 | 132.8 | 0.720 |
| 8 | 118.911 | 29.533 | 7 | 3728.0 | 6.276 | 0.897 | 0.801 | 1.634 | 0.147 | 103.6 | 0.770 |
| 9 | 118.950 | 29.561 | 8 | 5217.0 | 11.922 | 1.490 | 2.003 | 1.621 | 0.303 | 104.3 | 0.930 |
| 10 | 118.957 | 29.542 | 5 | 14829.7 | 126.369 | 25.274 | 1.298 | 1.970 | 0.289 | 135.2 | 0.710 |
| 11 | 118.981 | 29.612 | 4 | 4760.4 | 38.732 | 9.683 | 1.897 | 1.439 | 0.183 | 121.3 | 0.820 |
| 12 | 118.935 | 29.595 | 5 | 11219.5 | 99.969 | 19.994 | 2.170 | 1.560 | 0.460 | 125.0 | 0.830 |
| 13 | 119.100 | 29.541 | 5 | 5116.3 | 30.155 | 6.031 | 0.931 | 1.324 | 0.078 | 120.0 | 0.850 |
| 14 | 119.073 | 29.574 | 7 | 2281.6 | 3.303 | 0.472 | 1.157 | 1.399 | 0.177 | 94.7 | 0.930 |
| 15 | 119.080 | 29.541 | 5 | 1642.2 | 3.129 | 0.626 | 0.669 | 1.207 | 0.097 | 100.0 | 0.920 |
| 16 | 118.884 | 29.565 | 13 | 17962.3 | 76.655 | 5.897 | 3.277 | 1.720 | 0.534 | 107.7 | 0.810 |
| 17 | 118.844 | 29.492 | 7 | 3659.8 | 7.775 | 1.111 | 1.544 | 1.440 | 0.313 | 103.3 | 0.800 |
| 18 | 119.057 | 29.532 | 2 | 32625.9 | 870.295 | 435.147 | 1.410 | 2.290 | 0.475 | 271.0 | 0.248 |

Note：Landscape 1-18 were marked in Figure 1.
