# Supplementary material for: Positive effects of forest fragmentation per se on bryophyte diversity in subtropical fragmented forests: evidence from land-bridge islands
Source: Front Plant Sci. 2025 Apr 10;16:1539513. doi: 10.3389/fpls.2025.1539513 (PMC12018535; doi:10.3389/fpls.2025.1539513)
Supplement: Supplementary Table 4 — Species number of 14 bryophyte categories in 18 fragmented forest landscapes in the Thousand Island Lake (TIL). [file Table4.docx]

Table S4. Species number of 14 bryophyte categories in 18 fragmented forest landscapes in the Thousand Island Lake (TIL)

| Categories | Landscapes | | | | | | | | | | | | | | | | | |
| --- | --- | --- | --- | --- | --- | --- | --- | --- | --- | --- | --- | --- | --- | --- | --- | --- | --- | --- |
|  | 1 | 2 | 3 | 4 | 5 | 6 | 7 | 8 | 9 | 10 | 11 | 12 | 13 | 14 | 15 | 16 | 17 | 18 |
| Liverworts | 7 | 10 | 9 | 11 | 6 | 8 | 6 | 2 | 7 | 12 | 9 | 2 | 4 | 2 | 2 | 8 | 3 | 12 |
| Anomodontaceae | 3 | 3 | 3 | 5 | 2 | 3 | 1 | 1 | 2 | 4 | 2 | 1 | 1 | 1 | 2 | 3 | 1 | 5 |
| Brachytheciaceae | 1 | 7 | 4 | 5 | 5 | 6 | 5 | 0 | 3 | 11 | 10 | 1 | 1 | 0 | 0 | 4 | 1 | 8 |
| Bryaceae | 6 | 5 | 2 | 10 | 1 | 6 | 8 | 2 | 5 | 9 | 7 | 1 | 3 | 1 | 1 | 4 | 2 | 6 |
| Entodontaceae | 8 | 7 | 4 | 8 | 4 | 2 | 7 | 2 | 3 | 6 | 6 | 1 | 1 | 1 | 1 | 6 | 1 | 5 |
| Fissidentaceae | 7 | 6 | 6 | 8 | 4 | 3 | 5 | 3 | 4 | 7 | 5 | 3 | 3 | 3 | 3 | 4 | 2 | 8 |
| Hypnaceae | 10 | 11 | 7 | 12 | 8 | 5 | 10 | 5 | 11 | 10 | 9 | 4 | 8 | 7 | 4 | 9 | 4 | 8 |
| Leskeaceae | 2 | 4 | 4 | 4 | 2 | 0 | 1 | 0 | 2 | 4 | 3 | 1 | 1 | 0 | 1 | 2 | 0 | 1 |
| Leucobryaceae | 4 | 5 | 4 | 6 | 3 | 3 | 5 | 2 | 5 | 4 | 5 | 5 | 3 | 3 | 3 | 4 | 3 | 6 |
| Mniaceae | 2 | 2 | 2 | 3 | 1 | 4 | 2 | 2 | 3 | 6 | 2 | 0 | 1 | 0 | 1 | 2 | 1 | 3 |
| Orthotrichaceae | 1 | 5 | 3 | 4 | 3 | 0 | 3 | 2 | 1 | 4 | 2 | 0 | 0 | 1 | 1 | 2 | 0 | 4 |
| Pottiaceae | 11 | 13 | 7 | 14 | 6 | 6 | 10 | 5 | 12 | 12 | 8 | 2 | 4 | 4 | 3 | 10 | 1 | 11 |
| Thuidiaceae | 6 | 6 | 5 | 7 | 6 | 3 | 6 | 2 | 4 | 6 | 4 | 1 | 5 | 2 | 1 | 5 | 2 | 5 |
| Other moss families | 16 | 24 | 15 | 24 | 10 | 12 | 17 | 9 | 12 | 20 | 20 | 9 | 16 | 7 | 8 | 15 | 5 | 18 |
| Total | 84 | 108 | 75 | 121 | 61 | 61 | 86 | 37 | 74 | 115 | 92 | 31 | 51 | 32 | 31 | 78 | 26 | 100 |
