# Supplementary material for: Positive effects of forest fragmentation per se on bryophyte diversity in subtropical fragmented forests: evidence from land-bridge islands
Source: Front Plant Sci. 2025 Apr 10;16:1539513. doi: 10.3389/fpls.2025.1539513 (PMC12018535; doi:10.3389/fpls.2025.1539513)
Supplement: Supplementary Table 5 — Results of redundancy analysis on relationships of bryophyte SR with environmental variables in the TIL, summarizing eigenvalues, explained variation and additional statistics for each of the four ordination axes. Total variation is 1363.0000, explanatory variables account for 53.5% (adjusted explained variation is 31.1%. Test of significance of first canonical axis: pseudo-F = 9.8, P = 0.082. Test of significance of all canonical axes: pseudo-F = 21, P = 0.072. [file Table5.docx]

Table S5. Results of Redundancy Analysis on relationships of bryophyte SR with environmental variables in the TIL, summarizing eigenvalues, explained variation and additional statistics for each of the four ordination axes

| Statistic parameters | Axes | | | |
| --- | --- | --- | --- | --- |
|  | 1 | 2 | 3 | 4 |
| Eigenvalues | 0.4951 | 0.0534 | 0.021 | 0.0101 |
| Explained variation (cumulative) | 49.5100 | 54.8500 | 56.9500 | 57.9700 |
| Pseudo-canonical correlation | 0.8114 | 0.7502 | 0.7248 | 0.8453 |
| Explained fitted variation (cumulative) | 83.2800 | 92.2500 | 95.7900 | 97.5000 |

Note: Total variation is 1363.0000, explanatory variables account for 53.5% (adjusted explained variation is 31.1%.

Test of significance of first canonical axis: pseudo-F = 9.8, *P* = 0.082

Test of significance of all canonical axes: pseudo-F = 21, *P* = 0.072
