# Supplementary material for: Positive effects of forest fragmentation per se on bryophyte diversity in subtropical fragmented forests: evidence from land-bridge islands
Source: Front Plant Sci. 2025 Apr 10;16:1539513. doi: 10.3389/fpls.2025.1539513 (PMC12018535; doi:10.3389/fpls.2025.1539513)
Supplement: Supplementary Table 6 — Results of canonical correspondence analysis on relationships between bryophyte SC and environmental variables in the TIL, summarizing eigenvalues, explained variation and additional statistics for each of the four ordination axes. Total variation is 1.3851, explanatory variables account for 45.9% (adjusted explained variation is 8.0%. Test of significance of first canonical axis: pseudo-F = 1.2, P = 0.01. Test of significance of all canonical axes: pseudo-F = 1.1, P = 0.004. [file Table6.docx]

Table S6. Results of Canonical Correspondence Analysis on relationships between bryophyte SC and environmental variables in the TIL, summarizing eigenvalues, explained variation and additional statistics for each of the four ordination axes

| Statistic parameters | Axes | | | |  |
| --- | --- | --- | --- | --- | --- |
|  | 1 | 2 | 3 | 4 | |
| Eigenvalues | 0.1429 | 0.1128 | 0.0985 | 0.0877 | |
| Explained variation (cumulative) | 10.32 | 18.46 | 25.57 | 31.9 | |
| Pseudo-canonical correlation | 0.9921 | 0.9568 | 0.9765 | 0.9631 | |
| Explained fitted variation (cumulative) | 22.48 | 40.22 | 55.71 | 69.5 | |

Note: Total variation is 1.3851, explanatory variables account for 45.9% (adjusted explained variation is 8.0%

Test of significance of first canonical axis: pseudo-F = 1.2, *P* = 0.01

Test of significance of all canonical axes: pseudo-F = 1.1, *P* = 0.004
